# Enhanced Hydrolysis of Cellulose in Ionic Liquid Using Mesoporous ZSM-5

**DOI:** 10.3390/molecules23030529

**Published:** 2018-02-27

**Authors:** Tianlu Chen, Chunrong Xiong, Yousheng Tao

**Affiliations:** 1 CAS Key Laboratory of Design and Assembly of Functional Nanostructures, Haixi Institutes, Chinese Academy of Sciences (CAS), Fuzhou 350002, China; chentianlu@fjirsm.ac.cn; 2 College of Materials and Chemical Engineering, Hainan University, Haikou 570228, China

**Keywords:** mesopores, zeolites, ZSM-5, cellulose, catalysis, hydrolysis, ionic liquids

## Abstract

Mesoporous ZSM-5 prepared by alkaline treatment was demonstrated as an efficient catalyst for the cellulose hydrolysis in ionic liquid (IL), affording a high yield of reducing sugar. It was demonstrated that mesoporous ZSM-5 (SiO_2_/Al_2_O_3_ = 38) had 76.2% cellulose conversion and 49.6% yield of total reducing sugar (TRS). In comparison, the conventional ZSM-5 had a mere 41.3% cellulose conversion with 33.2% yield of TRS. The results indicated that the important role of mesopores in zeolites in elevating the TRS yield may be due to the diffusional alleviation of cellulose macromolecules. The effects of reaction time, temperature, and the ratio of catalyst to cellulose were investigated for optimal reaction conditions. It was found that IL could enter the inner channel of mesoporous ZSM-5 to promote the generation of H^+^ from Brönsted acid sites, which facilitated hydrolysis. Moreover, the mesoporous ZSM-5 showed excellent reusability for catalytic cycles by means of calcination of the used one, promising for its practical applications in the hydrolysis of cellulose.

## 1. Introduction

The conversion of renewable biomass into useful chemicals is of importance in green and sustainable chemistry [1,2,3]. Cellulose is the most abundant source of biomass. Its robust structure, composed of 1,4-β-glycosidic bonds of d-anhydroglucopyranose, has impeded its wide utilization [4,5]. Nevertheless, breakage of 1,4-β-glycosidic bonds by acids to hydrolyze cellulose into reducing sugar is a key process for the use of cellulose.

Efforts with mineral acids [6], enzymes [7], and supercritical water [8] have been devoted to cellulose hydrolysis. However, there are distinct drawbacks in these traditional methods, such as the corrosion of reactors, the high cost of enzymes, harsh reaction conditions, and difficult separation of the products [9,10]. On the contrary, solid acid catalysts have advantages of easy product separation, recyclability, and little damage of the reactor [11,12,13,14,15,16].

Due to containing both Lewis and Brönsted acid sites, zeolites have applications in the hydrolysis of cellulose [17,18,19,20,21]. Onda et al. [21] reported different H-form zeolites, such as H-mordenite, H-beta, and H-ZSM-5 for the hydrolysis of cellulose in water. Compared to sulfonated activated carbon, the product yield over H-ZSM-5 was low due to the small channels and cavities of zeolites, which imposed diffusional limitations on reactions. In order to improve the catalytic activities of zeolites under milder conditions, Cai et al. [17] proposed an approach by using an ionic liquid as the solvent to increase the solubility of cellulose. In this manner, the contact between cellulose and zeolites was improved. It became clear that the pore sizes and the acid amount of zeolites were critical for the high hydrolysis efficiency of cellulose.

Mesoporous zeolites have been applied in industry for fluid catalytic cracking and hydrocracking [22,23]. In recent years, mesoporous zeolites also have attracted a great interest in biomass hydrolysis owing to their high thermal stability and the diffusional alleviation of large reactant molecules [24,25,26]. Zhou et al. [25] used the hierarchical H-USY zeolites prepared by oxalic acid treatment for the hydrolysis of hemicelluloses. The yield of total reducing sugars (TRS) increased remarkably over H-USY of mesopores/macropores compared with conventional H-USY under the same reaction conditions. They also investigated sulfonated hierarchical H-USY for microcrystalline cellulose hydrolysis but achieved a mere 15.8% yield of TRS [26]. This suggests that although the mesopores of zeolites are favorable for the higher yield of sugars in hemicelluloses hydrolysis, it is a challenge to develop efficient catalytic hydrolysis of cellulose with zeolites. In this work, mesoporous ZSM-5 prepared with alkaline treatment was applied in the hydrolysis of cellulose in an ionic liquid of 1-butyl-3-methyl imidazolium chloride ([BMIm]Cl). We investigated the reaction conditions and catalyst reusability and demonstrated the role of mesopores in elevating the yield of TRS.

## 2. Experimental

### 2.1. Materials

Microcrystalline cellulose with an average particle size of 25 μm and the ionic liquid of 1-butyl-3-methylimidazolium chloride ([BMIm]Cl) were purchased from Adamas Organics, Shanghai, China. ZSM-5 (SiO_2_/Al_2_O_3_ = 38) and ZSM-5 (SiO_2_/Al_2_O_3_ = 100) were supplied by XFNANO Materials Tech Co., Ltd., Nanjing, China and Acros Organics, Geel, Belgium, respectively.

### 2.2. Characterization Methods

The powder X-ray diffraction (XRD) patterns were acquired on a MiniFlex II X-ray diffractometer (Rigaku Co., Salem, NH, USA) using Cu Kα (λ = 0.154050 nm) radiation. N_2_ adsorption and desorption isotherms on samples were measured at 77 K using a sorptometer ASAP2020 (Micromeritics, Norcross, GA, USA). Samples were evacuated at 10^−4^ Pa and 393 K for 2 h before the measurement. Al concentrations in the zeolites were determined by ICP-AES in an Ultima2 spectrometer (HORIBA JobinYvon, Paris, France). The Brönsted acid sites in the catalysts were measured by the titration method as follows: 20 mL of 0.01 M sodium hydroxide aqueous solution was added to 0.04 g of catalyst. The mixture was stirred for 2 h at room temperature. After centrifugal separation, the supernatant solution was titrated with a 0.01 M hydrochloric acid aqueous solution using phenolphthalein [21]. Elemental analysis of ionic liquid treated zeolite was done using a Viario MICRO elemental analyzer (Elementar, Langenselbold, Germany).

### 2.3. Preparation of Mesoporous ZSM-5

Mesorporous ZSM-5 was prepared with alkaline treatment. In a typical procedure, the parent ZSM-5 zeolites were treated in 0.2 M aqueous sodium hydroxide solutions at 338 K for different periods of time. The remaining solid product was filtered, washed, and dried at 373 K for 12 h. The alkaline-treated samples were converted into the H-forms by ion exchanged twice in a 1.0 M NH_4_NO_3_ solution at 353 K for 4 h, followed by calcination at 823 K for 5 h. The samples were denoted as H-Z-T, where T represented the time of alkaline treatment.

### 2.4. Catalytic Reactions

The hydrolysis of cellulose was performed in a sealed glass tube using the established procedures [17,24]. Fifty milligrams of cellulose was added portion wise to 1.0 g of [BMIm]Cl at 373 K. The mixture was stirred until a yellowish solution formed. To this solution were quickly added 0.2 mL of H_2_O and 50.0 mg of solid acid catalyst. The reaction mixture was taken out, weighed, quenched in ice water, and then centrifuged. The volumes of the samples were measured and the TRS of the products was analyzed. To test reusability of mesoporous zeolites, the solid residue was calcinated at 823 K for 5 h before use in the subsequent runs.

### 2.5. Analysis Methods

The conversion of cellulose was determined based on the differences in weight of cellulose before and after the reaction. The TRS of the products was analyzed using an established method [27]. The mixture containing 1.5 mL 3,5-dinitrosalicylic acid reagent and 0.2 mL samples was heated at 373 K for 5 min, then cooled to room temperature and diluted to 10 mL. The absorbance of the mixture was measured at 540 nm using a 1100 UV-VIS spectrophotometer (MAPADA Instruments Co. Ltd., Shanghai, China). The TRS concentration of the products was determined by using the adsorption factor of standard glucose solution. The mass of TRS and the yield of TRS from cellulose were calculated as follows:

*M*_T_ = TRS concentration (mg/mL) × 10 (mL) × (V/0.2) × (M_0_/M_1_)

TRS yield = *M*_T_/M × 100%

where *M*_T_ is the mass of TRS. V is the volume of the samples. M_0_ is the total mass of the reaction solution. M_1_ and M are the masses of samples and cellulose initially loaded for the reaction, respectively.

## 3. Result and Discussion

### 3.1. Chemical and Pore Structures of the Samples

**Figure 1:**
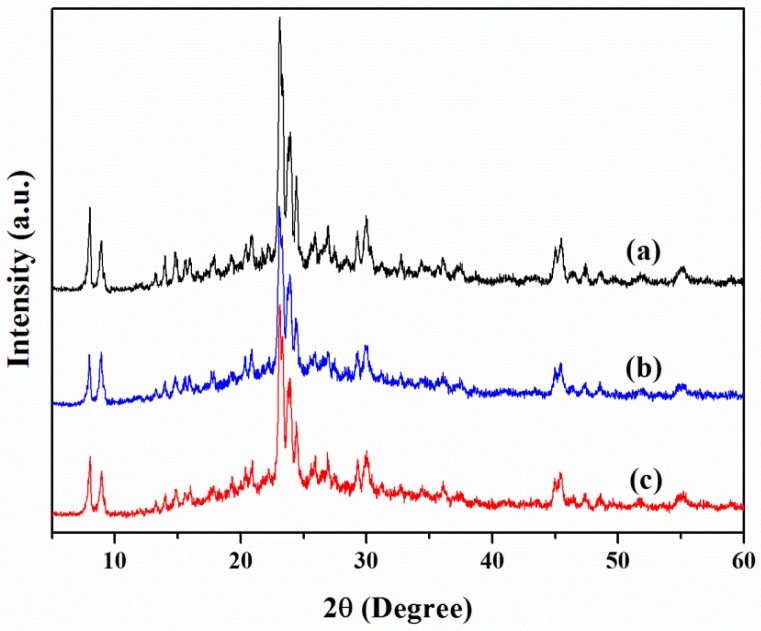
XRD patterns of the alkaline-treated ZSM-5 and the parent ZSM-5 zeolites: (**a**) H-Z-0; (**b**) H-Z-20; and (**c**) H-Z-30.

Alkaline treatment of ZSM-5 can create mesopores through the selective extraction of framework Si atoms, meanwhile, preserving its hydrothermal stability and acidity to some extent [28,29,30]. The XRD patterns of the parent ZSM-5 and the alkaline-treated ZSM-5 are shown in Figure 1. The basic fingerprints of zeolite after alkaline treatment were maintained, suggesting that the crystalline phases of zeolite did not change upon the alkaline treatment. However, the crystallinity of the samples decreased with the increasing period of alkaline treatment because the removal of Si from the framework of the zeolite formed large pores, as well as defect domains [31]. The FT-IR spectra of the parent ZSM-5 and the alkaline-treated ZSM-5 were also studied. The absorption bands of them were identical with respect not only to the band positions, but also to the peak intensity, suggesting the surface chemistry in alkaline treated ZSM-5 zeolites has remained virtually unchanged.

**Figure 2:**
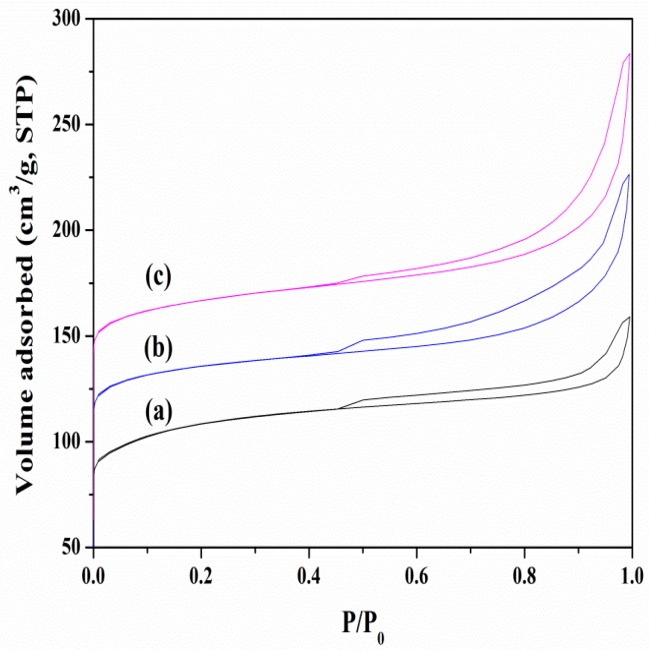
Adsorption/desorption isotherms of nitrogen at 77 K on the alkaline-treated ZSM-5 and the parent ZSM-5 zeolites: (**a**) H-Z-0; (**b**) H-Z-20; and (**c**) H-Z-30. For clarity the isotherms of H-Z-20 and H-Z-30 have been shifted upward by 50 cm^3^/g and 100 cm^3^/g, respectively.

Figure 2 shows the N_2_ adsorption/desorption isotherms at 77 K on the alkaline-treated ZSM-5 and the parent ZSM-5. Compared with that of parent zeolites, the isotherms of alkaline-treated zeolites had increases in uptakes and changes in the shapes of hysteresis loops extending from P/P_0_ = 0.4 to ~1, indicating that the mesopores had been created upon the alkaline treatments. The mesopores with pore sizes of 2–25 nm were gradually developed with the increase of the treatment period. Both the total pore volume and mesopore volume increased after the alkaline treatments, while the micropore volume was almost maintained, as displayed in Table 1. It was known that the amount of Al atoms was proportional to the amount of Brönsted acid sites [32]. The acid sites of H-Z-0, H-Z-20, and H-Z-30 zeolites were 0.43 mmol/g, 0.45 mmol/g, and 0.51 mmol/g, respectively. The higher acidity of mesoporous ZSM-5 in comparison with parent ZSM-5 was attributed to a decrease in the Si/Al ratio by the removal of framework Si [24].

### 3.2. Catalytic Performance of the Samples

#### 3.2.1. Hydrolysis of Cellulose

**Figure 3:**
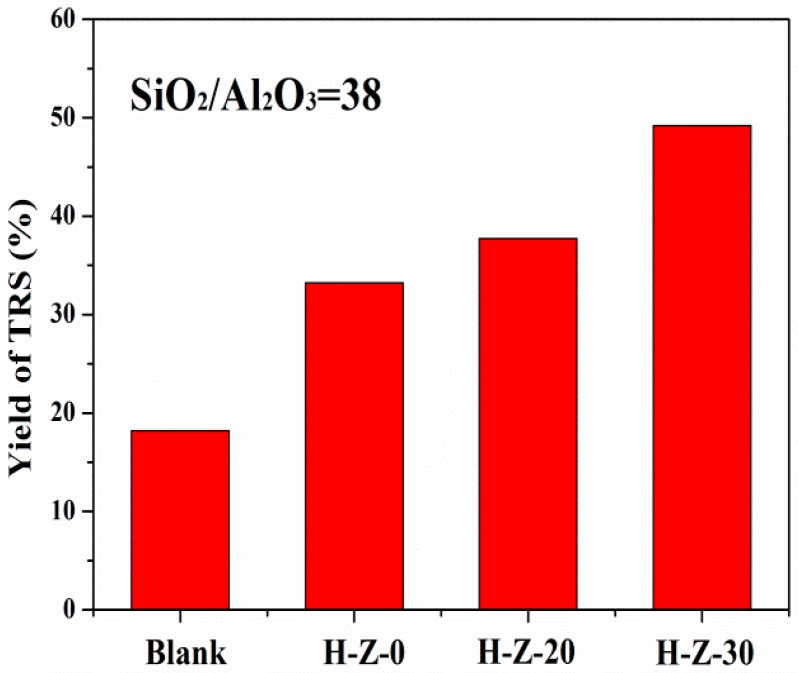
Hydrolysis of cellulose catalyzed by the alkaline-treated ZSM-5 and the parent ZSM-5 zeolites with SiO_2_/Al_2_O_3_ ratio of 38. Reaction conditions: [BMIm]Cl (1.0 g), cellulose (50.0 mg), catalyst (50.0 mg), water (0.2 mL), at 403 K and 2 h.

**Figure 4:**
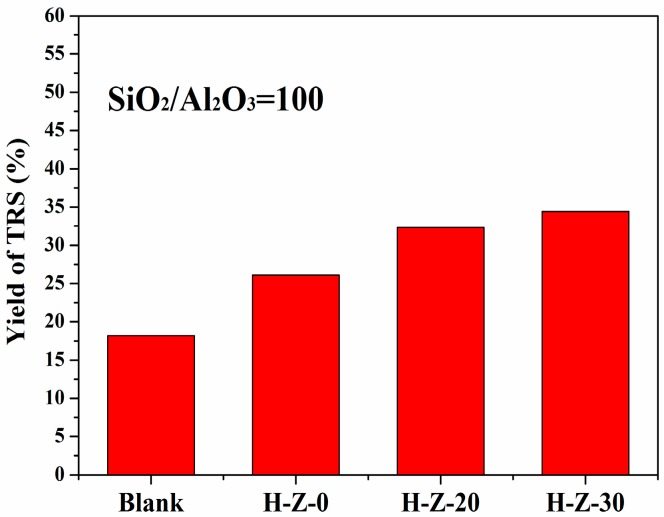
Hydrolysis of cellulose catalyzed by the alkaline-treated ZSM-5 and the parent ZSM-5 zeolites with SiO_2_/Al_2_O_3_ ratio of 100. Reaction conditions: [BMIm]Cl (1.0 g), cellulose (50.0 mg), catalyst (50.0 mg), water (0.2 mL), at 403 K and 2 h.

Pore structures and acidity are the important factors affecting the performance of the catalysts for the cellulose hydrolysis. Figure 3 and Figure 4 show the hydrolysis of cellulose in [BMImCl] catalyzed by the alkaline-treated ZSM-5 and the parent ZSM-5 zeolites with SiO_2_/Al_2_O_3_ ratios of 38 and 100, respectively. As a reference reaction, cellulose hydrolysis in IL without catalyst had a TRS yield of 18.2%. The cellulose can dissolve in IL to form a homogeneous solution. The dissociated Cl^-^ and the electron-rich aromatic π system of [BMIm]^+^ in [BMIm]Cl should weaken the glycosidic linkage of cellulose to facilitate hydrolysis [33,34].

When the parent ZSM-5 (SiO_2_/Al_2_O_3_ = 38) was used as a catalyst to hydrolyze cellulose at 403 K for 2 h, it gave the cellulose conversion of 41.3% with a TRS yield of 33.2%. In contrast, the corresponding values increased remarkably with the use of the alkaline-treated ZSM-5. Mesopores made a large number of the acid sites of zeolites accessible to the β-glucosidic bonds, promoting the mass transfer of cellulose and reducing sugars in a timely manner [35]. The mesopores also facilitated the [BMIm]Cl entering the inner spaces of the zeolites. Ion-exchange involving [BMIM]^+^ and H^+^ species promoted the generation of H^+^ from Brönsted acid sites of H-form zeolites [17,36]. The higher acidity or the larger amount of Brönsted acid of mesoporous ZSM-5 than that of parent ZSM-5 due to its lower Si/Al ratio also contributed to higher activity in the cellulose hydrolysis [37,38]. H-Z-30 zeolite had excellent catalytic performance, with a maximum of 76.2% cellulose conversion and 49.6% TRS yield. The large amount of mesopores of H-Z-30 zeolite facilitated the reaction products to diffuse out the catalyst instead of their further conversion to byproducts.

Similarly, hydrolysis of cellulose was studied using parent ZSM-5 and mesoporous ZSM-5 zeolites with the ratio of Si/Al (SiO_2_/Al_2_O_3_ = 100). The yield of TRS increased to 34.4% over mesoporous H-Z-30 (100) in comparison with 26.4% yield of TRS over H-Z-0 (100). However, it was found that the TRS yield decreased with the increase of the Si/Al ratio due to the fewer acid sites.

#### 3.2.2. Effect of Reaction Conditions on Cellulose Hydrolysis over H-Z-30

**Figure 5:**
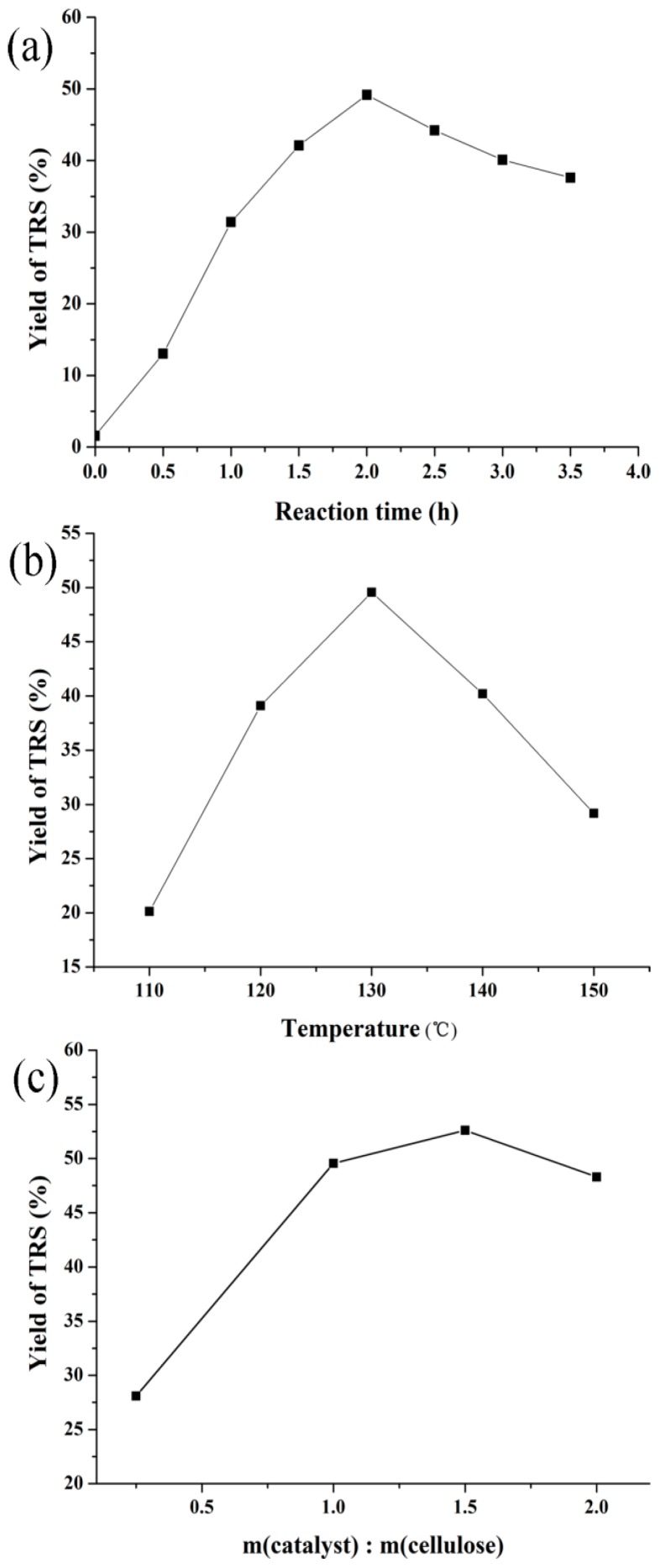
The effect of reaction time (**a**); temperature (**b**); and weight ratio of catalysts to cellulose (**c**) on TRS yields over H-Z-30. Reaction conditions: [BMIm]Cl (1.0 g), water (0.2 mL).

The effect of reaction conditions such as reaction time, temperature, and the ratio of catalyst and cellulose on the catalytic performance of H-Z-30 were investigated in order to maximize the yield of TRS. The reaction time is important for the cellulose hydrolysis due to the fact that sugars can be further transformed to other byproducts, such as levulinic acid, 5-hydroxymethylfurfural (5-HMF), formic acid, and furfural [39]. The effect of reaction time of H-Z-30 on TRS yields was investigated at 403 K. As shown in Figure 5a, with the increase of reaction time, the yield of TRS increased obviously at the initial stage. After reaching the maximum yield of 49.6% in 2 h, the TRS yield slowly reduced from 2.5 h to 3.5 h owing to the conversion of the formed sugars. Figure 5b presents the effect of reaction temperature on TRS yields. The yield of TRS increased from 20.1% to 49.6% as the temperature was elevated from 383 K to 403 K. Subsequently, the TRS yield decreased with the increasing temperature because the produced sugars decomposed to 5-HMF easily at high temperature [18,24,40]. After the reaction the solid became black due to insoluble side-products containing humins at 423 K. Figure 5c demonstrates the effects of the weight ratio of catalyst to cellulose on the TRS yield. The TRS yield increased and then decreased with the increase of the catalyst usage. The highest yield of TRS (52.6%) was obtained at the weight ratio of catalyst to cellulose being 1.5. After that, catalysts had redundant acid sites which accelerated the transformation of the produced sugars to other byproducts.

#### 3.2.3. The Role of Mesopores in Zeolites for Ionic Liquid

**Figure 6:**
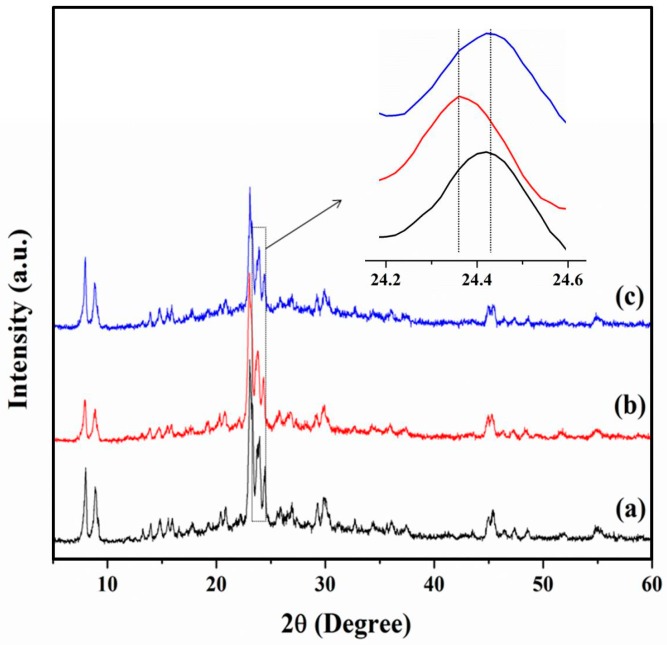
XRD patterns of H-Z-30 before and after IL-treatments. (**a**) H-Z-30; (**b**) IL-treated H-Z-30; and (**c**) samples of calcined IL-treated H-Z-30 at 823 K for 5 h.

**Figure 7:**
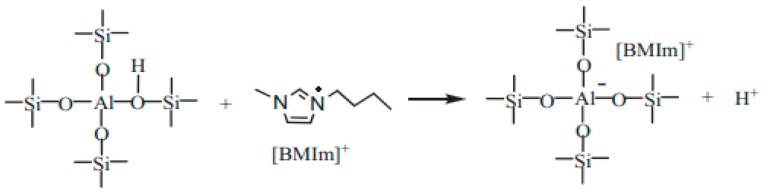
The proposed reaction pathway of zeolite and [BMIm]^+^.

Previous work showed that the IL could enter the inner channel of H-form zeolites to promote cellulose hydrolysis [9,17,33,40]. In order to illustrate the mutual interactions of mesoporous ZSM-5 and IL during the hydrolysis process, IL-treated H-Z-30 was characterized by means of XRD, as shown in Figure 6. The XRD patterns of fresh H-Z-30 (a), the IL-treated H-Z-30 (b), and the recovered H-Z-30 after calcinations demonstrated that the framework structure of H-Z-30 was particularly stable in IL. However, the peak at 24.4° of IL-treated H-Z-30 shifted about 0.09° to lower angle value with respect to fresh catalyst, indicating that [BMIm]Cl entered the pore of H-Z-30 due to the dilatation effect. The element analysis of IL-treated H-Z-30 indicated N and C mass contents of 1.68% and 6.49% (the N/C molar ratio was 1:4), matching with the N/C molar ratio in [BMIm]^+^ ([C_8_H_15_N_2_]^+^). The N_2_ adsorption results further demonstrated that IL entered and occupied some nanospace in H-Z-30. The BET surface area, mesopore volume, and total pore volume of fresh H-Z-30 were determined to be 350 m^2^/g, 0.22 cm^3^/g, and 0.34 cm^3^/g, respectively (Table 1). Comparatively, the corresponding values of IL-treated H-Z-30 decreased to 70 m^2^/g, 0.19 cm^3^/g, and 0.20 cm^3^/g, respectively. After calcinations, the values recovered to 350 m^2^/g, 0.22 cm^3^/g, and 0.34 cm^3^/g, respectively. This suggested that the IL in the pores of H-Z-30 could be removed by calcination. Hence, the [BMIm]^+^ entered the inner channels and caves of mesoporous ZSM-5 and promoted the generation of H^+^ from Brönsted acid sites via ion exchange to facilitate hydrolysis. The proposed reaction pathway of zeolite and [BMIm]^+^ is shown in Figure 7.

#### 3.2.4. Catalytic Cycling of Mesoporous ZSM-5 in Cellulose Hydrolysis

**Figure 8:**
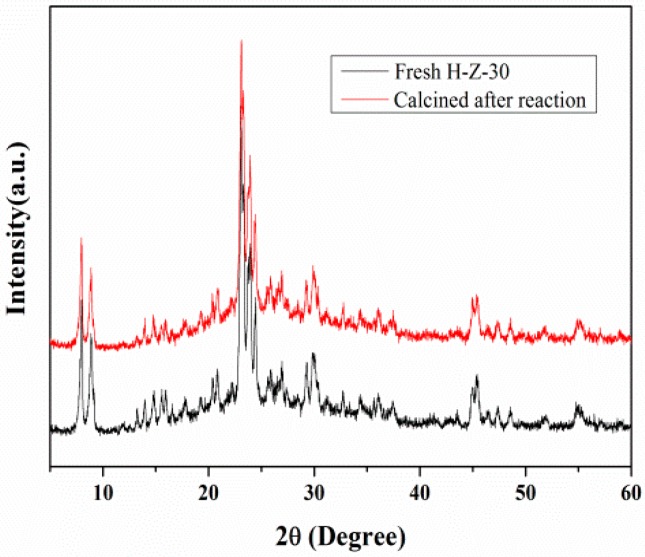
XRD patterns of fresh H-Z-30 and calcined H-Z-30 after the cellulose hydrolysis.

**Figure 9:**
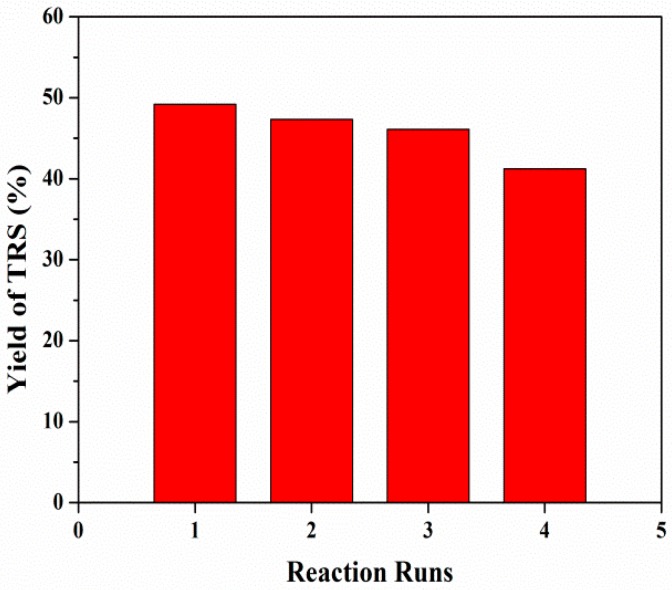
Yield of TRS vs. catalytic cycles of H-Z-30 in hydrolysis of cellulose. Reaction conditions: [BMIm]Cl (1.0 g), cellulose (50.0 mg), catalyst (50.0 mg), water (0.2 mL), at 403 K and 2 h.

The catalytic activity and stability of zeolite were important for its practical applications in the hydrolysis of cellulose. After each reaction, H-Z-30 was separated for the calcination. After each cycle as well as calcination, the crystalline phases of mesoporous ZSM-5 were identical to the fresh one (Figure 8) and the acidity sites remained (0.49 mmol/g). As shown in Figure 9, H-Z-30 was stable for three catalytic cycles, with a 41.3% yield of TRS.

## 4. Conclusions

Mesoporous ZSM-5 zeolites prepared by alkaline-treatment of parent zeolites almost preserved their acidity and stability. The samples were applied in catalytic hydrolysis of cellulose in an ionic liquid to afford reducing sugars. The mesopores played an important role in elevating the yield of TRS. The mesopores not only improved the accessibility of the acid sites, but might also facilitate the reaction products to diffuse out of the catalysts in a timely manner, restraining its further conversion to byproducts. The role of diffusion on the reaction progress needs to be illustrated in future work. Moreover, the mesopores made [BMIm]^+^ enter the nanospaces of ZSM-5, promoting the generation of H^+^ from Brönsted acid sites via ion exchange to facilitate hydrolysis. On the other hand, the higher acidity of mesoporous zeolites (the lower Si/Al molar ratio) was also helpful for the hydrolysis of cellulose. Importantly, the mesoporous ZSM-5 showed excellent reusability for catalytic cycles, promising for its practical applications in the hydrolysis of cellulose.

## Figures and Tables

**Table 1:** The amount of Al atoms, pore structure parameters and the acid sites of the parent and mesoporous ZSM-5 zeolites.

Catalyst Sample	Al (wt %)	BET Surface Area (m^2^/g)	Pore Volume (cm^3^/g)			Acid Sites (mmol/g)
			V_meso_	V_micro_	V_total_	
H-Z-0	1.95	365	0.13	0.13	0.26	0.43
H-Z-20	2.03	340	0.18	0.13	0.31	0.45
H-Z-30	2.36	350	0.22	0.12	0.34	0.51
